# Insects as Stem Engineers: Interactions Mediated by the Twig-Girdler *Oncideres albomarginata chamela* Enhance Arthropod Diversity

**DOI:** 10.1371/journal.pone.0019083

**Published:** 2011-04-19

**Authors:** Nancy Calderón-Cortés, Mauricio Quesada, Luis H. Escalera-Vázquez

**Affiliations:** 1 Centro de Investigaciones en Ecosistemas, Universidad Nacional Autónoma de México, Morelia, Michoacán, México; 2 Departamento de Zoología, Instituto de Biología, Universidad Nacional Autónoma de México, México D.F., México; Ghent University, Belgium

## Abstract

**Background:**

Ecosystem engineering may influence community structure and biodiversity by controlling the availability of resources and/or habitats used by other organisms. Insect herbivores may act as ecosystem engineers but there is still poor understanding of the role of these insects structuring arthropod communities.

**Methodology/Principal Findings:**

We evaluated the effect of ecosystem engineering by the stem-borer *Oncideres albomarginata chamela* on the arthropod community of a tropical dry forest for three consecutive years. The results showed that ecosystem engineering by *O. albomarginata chamela* had strong positive effects on the colonization, abundance, species richness and composition of the associated arthropod community, and it occurred mainly through the creation of a habitat with high availability of oviposition sites for secondary colonizers. These effects cascade upward to higher trophic levels. Overall, ecosystem engineering by *O. albomarginata chamela* was responsible for nearly 95% of the abundance of secondary colonizers and 82% of the species richness.

**Conclusions/Significance:**

Our results suggest that ecosystem engineering by *O. albomarginata chamela* is a keystone process structuring an arthropod community composed by xylovores, predators and parasitoids. This study is the first to empirically demonstrate the effect of the ecosystem engineering by stem-boring insects on important attributes of arthropod communities. The results of this study have important implications for conservation.

## Introduction

One of the central issues in ecology is to understand the mechanisms that structure ecological communities. Even though direct pairwise interactions (e.g. competition and predation) play a major role in explaining the structure of many biological communities (e.g. [1], [2]), other interactions can also be important [3], [4]. One interaction with important consequences on biological communities and biodiversity is the relationship between organisms that modify or create new habitats with those organisms that use these new habitats, a process called “ecosystem engineering” [5], or habitat modification [6]. Ecosystem engineers are species that control the availability of resources for other species by causing physical state changes in biotic or abiotic materials [5]. Because some ecosystem engineers create habitats where entire communities establish, they are also called “foundation species” [6] or “keystone engineers”, when the impact of the ecosystem engineer is higher than its abundance [7]. Currently, an increasing number of studies have experimentally demonstrated that some species act as ecosystem engineers (e.g. beavers, salmons, pocket-gophers) affecting communities and ecosystems (reviewed in [8], [9]).

Several insect herbivores manipulate their host-plants to build a variety of structures, which are secondarily occupied by organisms other than the original constructor. Hence, these herbivores can act as ecosystem engineers [10]. However, the role of insects as ecosystem engineers has only been experimentally evaluated for leaf-rollers [11], [12], gall makers [13] and leaf miners [14], [15]. These studies indicate that ecosystem engineering by insect herbivores influence overall abundance, species richness, and composition of arthropod communities by providing new habitats for other herbivores that are used for shelter (from natural enemies and adverse microclimates) and for more nutritious food [4], [10]–[15]. Ecosystem engineering effects can propagate to higher trophic levels, triggering cascades of interactions including trophic, antagonistic and mutualistic interactions [4], [11], [13].

One insect guild comparatively less studied in this regard is represented by stem-borers, which are insects that develop (for at least part of their life cycle) in wood, bark or woody stems of plants [16]. Many of them begin their life cycle as eggs laid under bark by free-living adult females; the larvae feed on the wood inside stems and eventually emerge as adults to repeat the cycle [16]. The stem-boring larvae produce complex systems of cavities that can be secondarily occupied by other arthropods [10], [17]–[20]. This suggests that the guild of stem-borers includes several species that can act as ecosystem engineers. However, empirical studies evaluating the effects of stem engineering on arthropod communities are currently lacking.

Stem-boring insects play important functional roles in forest ecosystems, as they contribute to nutrient cycling [21]–[23], alteration of tree architecture [23], [24], resource regulation [25], and alteration of the composition and hydrology of forests [25], [26]. Therefore, the study of factors structuring their communities has important implications for forest conservation.

Here we present the results of a field experiment designed to evaluate the effect of ecosystem engineering by the stem-boring beetle, *Oncideres albomarginata chamela* (Cerambycidae: Lamiinae), on the arthropod community associated with detached branches of *Spondias purpurea* (Anacardiaceae). The study was carried out for three consecutive years. *O. albomarginata chamela* actively manipulates its host plant through a process consisting of two steps. First, adult females of *O. albomarginata chamela* preferentially girdle and detach reproductive branches of *S. purpurea,* before the reproductive season of the tree [27], when reproductive branches have accumulated the maximum concentration of non-structural carbohydrates [28] and nitrogen [27]. Second, adult females make incisions and gnaw egg niches along the detached branches for oviposition [27]. Therefore, *O. albomarginata chamela* females provide a high quality environment for offspring development [29]. Incidentally, these females also provide a suitable environment for secondary colonization [30], particularly for insects that oviposit opportunistically in cracks and crevices in the bark or cortex of plants [16], [30].

Based on this evidence, we hypothesized that the modification of tree branches by *O. albomarginata chamela* plays a key role in the establishment of a new arthropod community and promotes interactions with positive effects on arthropod abundance and diversity. To test this hypothesis, we simulated *O. albomarginata chamela* physical modification of *S. purpurea* branches, and compared the community composition, frequency of colonization, abundance and species richness of secondary arthropod colonizers between non-engineered and engineered branches (both artificially and naturally modified branches).

## Methods

### Ethics statement

All animals were handled according to relevant national and international guidelines. Insects were reared at natural conditions at the study site, and released *in situ* after the experiment. The animal work was approved by the authorities of Chamela Biological Station, Universidad Nacional Autónoma de México (National Autonomous University of Mexico), and by national authorities of Secretaría de Medio Ambiente y Recursos Naturales (Secretary of Environment and Natural Resources; SEMARNAT, permission SGPA/DGVS/05876/10).

### Study system

*Oncideres albomarginata chamela* Chemsak and Gisbert is a longhorn beetle (Cerambycidae) that detaches branches 2–3 cm in diameter from the tropical tree *Spondias purpurea* L. (Anacardiaceae), and oviposits in them [27]. Alternative but less used host plants of *O. albomarginata chamela* include: *Comocladia engleriana* Loes (Anacardiaceae), *Mangifera indica* L. (Anacardiaceae), *Amphipterygium adstringens* Schide ex Schlecht (Rubiaceae), *Bursera* Jacq. ex L. spp. (Burseraceae), *Ceiba pentandra* (L.) Gaertn (Bombacaceae), *Urera* (L.) Gaud. sp. (Urticacaceae) and *Delonix regia* (Bojer ex Hook) Raf. (Fabaceae) [31]. *O. albomarginata chamela* is distributed in Mexico in the states of Jalisco, Nayarit, Guerrero, Oaxaca, Chiapas and Veracruz [32], but *O. albomarginata* Thomson is distributed in México, Central America (Nicaragua) and South America (British and French Guiana, Venezuela) [33]. The body length of *O. albomarginata chamela* is 17–31 mm and 6.5–12 mm wide [32]. The reproductive period of this species is from October to February; eggs hatch and larvae develop inside detached branches until the adults emerge in low densities 6–8 months later. Adult females of *O. albomarginata chamela* are the only herbivores at the study site that detach branches of *S. purpurea* and immediately oviposit in them [27], but after a certain period of time other species of stem-boring beetles (mainly non-girdling species) take advantage of the detached branches and oviposit in them as well.

*S. purpurea* is a common dioecious tree of the tropical dry forest of Mexico [28]. The ratio of male and female trees of *S. purpurea* in the population at the study site is 1∶1 [27]. This species can reach 15 m in height and almost 80 cm in diameter at the base; leaves are compound with 5 to 12 elliptic-acute leaflets from 2 to 4 cm in length [34]. Flowers are red, sessile, unisexual and dimorphic between males and females [34]. Trees are deciduous with flowering and fruiting occurring from December to May, and leaves are maintained from June to November [34].

### Study site

The study was conducted in the Chamela-Cuixmala Biosphere Reserve at Chamela Biological Station, UNAM (19°30′N, 105°03′W) located on the Pacific coast of Jalisco, Mexico, from December 2006 to January 2010. The vegetation is tropical dry forest with a mean annual rainfall of 707 mm and a dry season that extends from November to June [35].

### Experimental design

In order to evaluate the effect of ecosystem engineering by *O. albomarginata chamela* on the arthropod community, during December 2006 to January 2008, we conducted a field experiment consisting of three treatments (*N ≈* 50 branches/treatment): *O. albomarginata chamela* engineered and colonized-branches (OE), artificially simulated engineered branches (SE), and non-engineered branches (NE). For treatment OE, we collected branches of *S. purpurea* naturally detached and colonized by *O. albomarginata chamela* on December 2006. This treatment was used as control to provide baseline data on the arthropod community associated with *S. purpurea* branches, and to analyze the effects of the ecosystem engineer presence. For treatment SE, branches exhibiting similar characteristics (reproductive branches from 2–3 cm in diameter) to those detached and colonized by *O. albomarginata chamela* were artificially cut off from *S. purpurea* trees. We artificially simulated the structural modification of branches made by adult females of *O. albomarginata chamela*, by making numerous incisions with scissors (every 5 mm) on the bark of these branches. Treatment NE consisted of simply artificially detached reproductive branches of 2–3 cm in diameter of *S. purpurea* with no manipulation. We called this treatment “non-engineered branches” because mechanical factors, such as wind, water stress, mechanical branch damage, among others, detach a great proportion of branches and twigs from trees in the study site. Specifically, broken branches (2–20 cm in circumference) constitute the most important component (43%) of the forest total above-ground dead phytomass in Chamela tropical dry forest [36]. Thus, broken branches can represent non-engineered but available habitats. All branches were marked, and they were left hanging on the source-tree for 45 days (December 2006 to February 2007) to allow the colonization of secondary opportunistic species. Our preliminary analysis indicated that 30–45 days (during that period of the year) is when most insect borers colonize *S. purpurea* detached branches. The gender of each source-tree was registered. To control for the size of the branches used for each treatment, we measured the diameter at the point of branch cutting with an electronic caliper (Mytutoyo Inc). To control for adult female host selection, we cut off two branches for treatments NE and SE from the same tree where *O. albomarginata chamela* had previously detached and colonized branches. Additionally, the treatments were conducted in the same host plants to control for any related chemical attractive signals emitted by the same tree, as well as to control for any other factors associated with the nutritional value of host trees. Therefore, the branches of the three treatments had the same probability to be located by secondary colonizers. After 45 days, all branches were enclosed in mesh bags (<0.5 mm mesh size) to prevent further colonization and escape of the established fauna. Branches collected in mesh bags were placed in an open room at the study site, and maintained at local environmental conditions. Emerging arthropods from each branch were recorded monthly from March 2007 to January 2008, and released. We measured the total length of 20–40 adults of each insect species to estimate the size of the secondary colonizers. The exact same experiment was repeated for two more years: December 2007 to January 2009, and December 2008 to January 2010. Taxonomic identification of species that emerged was carried out by the beetle specialist Dr. Felipe A. Noguera and using available taxonomic literature [37]–[39].

### Data analysis

First, we compared the diameter (at the point of branch cutting) of detached branches to determine differences in sizes between treatments, through one way Analysis of Variance (ANOVA) using PROC ANOVA [40]. Our results indicated that branch diameter did not differ significantly between treatments (2007: F_2, 154_ = 1.564, P = 0.213; 2008: F_2, 155_ = 1.068, P = 0.346; 2009: F_2, 178_ = 0.263, P = 0.769). In a previous study, Uribe-Mú and Quesada [27] found that branch gender had no effect on *O. albomarginata chamela* larval performance. Therefore we expected that branch gender had no effect on the number of secondary colonizers emerging from *S. purpurea* branches. This was confirmed when we analyzed the variation associated with branch gender through a Generalized Linear Model using a GENMOD procedure [40], in which the number of secondary colonizers that emerged from *S. purpurea* branches was used as the response variable, and tree gender as the independent variable. We used a Poisson distribution with a logarithmic link function for the analysis and corrected for overdispersion of data. Tree gender had no significant effect (2007: χ^2^ =  0.37, P = 0.5428; 2008: χ^2^ = 1.42, P = 0.2327; 2009: χ^2^ =  2.09, P = 0.1479) and was not included in further analyses.

Non-metric multidimensional scaling (NMDS) was used as an ordination procedure to determine differences in community composition among OE, SE and NE branches. The NMDS analysis was based on ranked Bray-Curtis dissimilarity distances [41]. Differences in community composition between treatments were tested using an analysis of similarity (ANOSIM), which uses 1000 random reassignments of species to groups and determines whether the group assignments were significantly different from those generated by chance. NMDS and ANOSIM analyses were performed with the software PRIMER 5.2.9 for windows (PRIMER-Ltd, Plymouth, U.K.). Multiple comparisons in ANOSIM were made using a sequential Bonferroni correction [42].

To evaluate the effect of *O. albomarginata chamela* on the frequency of colonization of *S. purpurea* branches by secondary colonizers, each species was quantified as being present or absent. Data were analyzed using a Generalized Linear Mixed Model that implements a generalization of the standard linear model allowing the incorporation of random effects [43]. We used the GLIMMIX procedure in SAS statistical software with a binomial distribution, and a logit link function specified for the dependent variable [40]. Branch condition (colonized *vs*. non-colonized) was the response variable. Treatment, year and their interaction were included as fixed variables, while tree identity and its interaction with treatment as random effects. We used a Least Square Means (LSMeans) test for *a posteriori* comparisons [40].

Secondary xylovores showed two general traits in size and developmental time. These are key life-history traits in insects related to fitness, habitat selection, oviposition strategies, and response to natural enemies [44]. Therefore, we used them to define two putative life forms: a) species with small body size and short developmental time (life form I); and b) species with large body size and longer developmental time (life form II). Natural enemies were analyzed separately. A Generalized Linear Mixed Model was used to evaluate the effect of *O. albomarginata chamela* on the abundance of the secondary colonizers (SAS, GLIMMIX procedure) [40]. This model used: (i) the number of adult secondary colonizers that emerged from branches as the response variable, (ii) treatment, year and their interaction as fixed variables, and (iii) plant identity and its interaction with treatment as random effects. We used a Poisson distribution with a logarithmic link function in the analysis. The degrees of freedom of F-tests for the fixed effects were adjusted using the Satterthwaite method. To control for overdispersion, we applied a Poisson error distribution to the model. We used LSMeans tests for *a posteriori* comparisons [40].

To determine the impact of ecosystem engineers on species richness of engineered-habitats, we used a Generalized Linear Mixed Model (SAS, GLIMMIX procedure) [40]. We used the same model applied for the abundance analysis, but in this case the number of species that emerged from *S. purpurea* branches was the response variable. An increased number of species is expected as a random consequence of larger pools of individuals [45]. Therefore, to examine whether treatment differences in the species richness of secondary colonizers were driven by differences in the abundance of secondary colonizers, we constructed rarefaction curves for each treatment. We used cumulative species per branch including all branches sampled during the three study years (EcoSim 7.0, 10,000 iterations) [46].

## Results

### Effect of habitat engineering on community composition

In total, 28,301 secondary colonizers emerged from 478 detached branches of *S. purpurea* in three consecutive years of study. These included at least 25 species from eight families (Table 1), of which Bostrichidae (Coleoptera) was the most abundant, comprising 76% (±10 SD) of the overall natural arthropod community (Table 1), and Cerambycidae was the most diverse (9 spp.; Table 1). The natural arthropod community consisted of xylovore and predatory beetles, and parasitic wasps (Table 1). In addition to secondary colonizers that use *S. purpurea* branches for oviposition and offspring development, other “inquiline” species (which eventually arrived to *S. purpurea* branches, but did not oviposit in them) were recorded. These species included: termites, ants, pseudoscorpions, spiders, crickets and silverfish. However, given that inquilines emerged in very low numbers and were not present every year, we did not consider them in further analyses.

**Table 1:** Secondary colonizers that emerged from *Spondias purpurea* branches detached and colonized by *Oncideres albomarginata chamela*.

Family	Abundance (%)	Species	Size (mm)
**XYLOVORE BEETLES**			
**Bostrichidae**	75.76 (±9.8)	*Amphicerus* (LeConte) sp. ‡	11.13 (±1.43)
		*Bostrychopsis* (Lesne) sp. †	3.58 (±0.23)
		*Dendrobiella* (Casey) sp. * †	5.49 (±0.25)
		*Melalgus* (Dejean) sp. ‡	11.56 (±1.42)
		*Micrapate* (Casey) sp. †	3.56 (±0.18)
		*Prostephanus truncatus* (Horn) †	3.40 (±0.22)
		*Xylobiops* (Casey) sp. †	3.71 (±0.24)
**Curculionidae**	4.10 (±3.1)	*Hypothenemus* (Weswoot) spp. †	1.59 (±0.27)
(Scolytinae)		*Pityophthorus* (Eichhoff) sp. †	2.00 (±0.25)
**Lyctidae**	7.07 (±3.7)	*Lyctus* (Fabricius) sp. †	3.11 (±0.38)
**Buprestidae**	1.45 (±1.2)	*Acmaeodera* (Eschscholtz) sp. †	6.27 (±0.54)
		*Agrilus* (Curtis) sp. †	4.43 (±0.35)
**Cerambycidae**	3.25 (±2.6)	*Ataxia alpha* (Chemsak and Noguera)* §	14.30 (±1.44)
		*Estoloides chamelae* (Chemsak and Noguera)* ‡ §	12.47 (±1.17)
		*Eutrichillus comus* (Bartes) ‡	8.08 (±0.47)
		*Lagocheirus obsoletus* (Thomson) ‡	13.52 (±1.53)
		*Lissonotus flavocinctus* (Dupont)* ‡ §	13.53 (±2.63)
		*Poliaenus hesperus* (Chemsak and Noguera) ‡	8.69 (±0.60)
		*Sphaenothecus maccartyi* (Chemsak and Noguera) ‡ §	14.61 (±1.46)
		*Sphaenothecus trilineatus* (Dupont) ‡	21.42 (±1.52)
		*Trachyderes mandibularis* (Serville)* ‡ §	21.97 (±0.79)
**NATURAL ENEMIES**			
Predator beteles			
**Histeridae**	7.58 (±3.2)	*Teretriosoma nigrescens* (Lewis) †	2.26 (±0.16)
**Cleridae**	0.30 (±0.2)	*Enoclerus quadrisignatus* (Say.) ‡	10.40 (±0.64)
Parasitic waps			
**Hymenoptera**	0.48 (±0.06)	ND §	ND

*Not recorded in 2007;

† Life form I;

‡ Life form II;

§ Not recorded in non-engineered branches (NE); ND = not determined. Abundance values are means across the three years (±SD).

Secondary xylovores of life form I (Table 1) began to emerge one month after branches were enclosed in mesh bags, with a maximum emergence peak recorded in May. Secondary xylovores of life form II and natural enemies (Table 1) emerged throughout the year, but their maximum emergence peaks were observed in September and July, respectively. *O. albomarginata chamela,* the species with the greatest size (23.58 mm ±2.24), was the last species to emerge (September to December). These emergence patterns were consistent across years.

**Figure 1:**
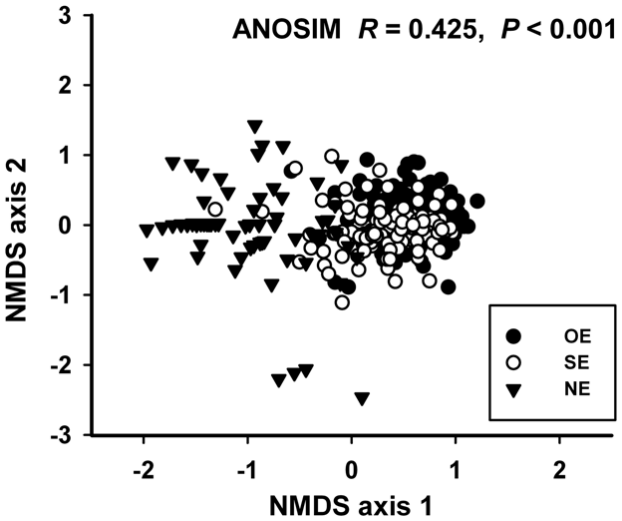
Arthropod community composition in detached *Spondias purpurea* branches. OE  =  *O. albomarginata chamela*-engineered branches; SE  = simulated-engineered branches; and NE  = non-engineered branches. Each point is a two-dimensional (axis 1 and axis 2) representation of the arthropod community composition on an individual branch based on global non-metric multidimensional scaling (NMDS) analysis (stress  = 0.19).

There were significant differences in the composition of the community of secondary colonizers between treatments (R = 0.425, n = 478, P<0.01; Figure 1). However, the strongest differences in community composition were between non-engineered (NE) and engineered (OE and SE) branches (NE vs. OE: R = 0.691, P<0.01; NE *vs.* SE: R = 0.574, P<0.01; SE vs. OE: R = 0.098, P<0.01).

### Effect of habitat engineering on branch colonization frequency by secondary colonizers

**Figure 2:**
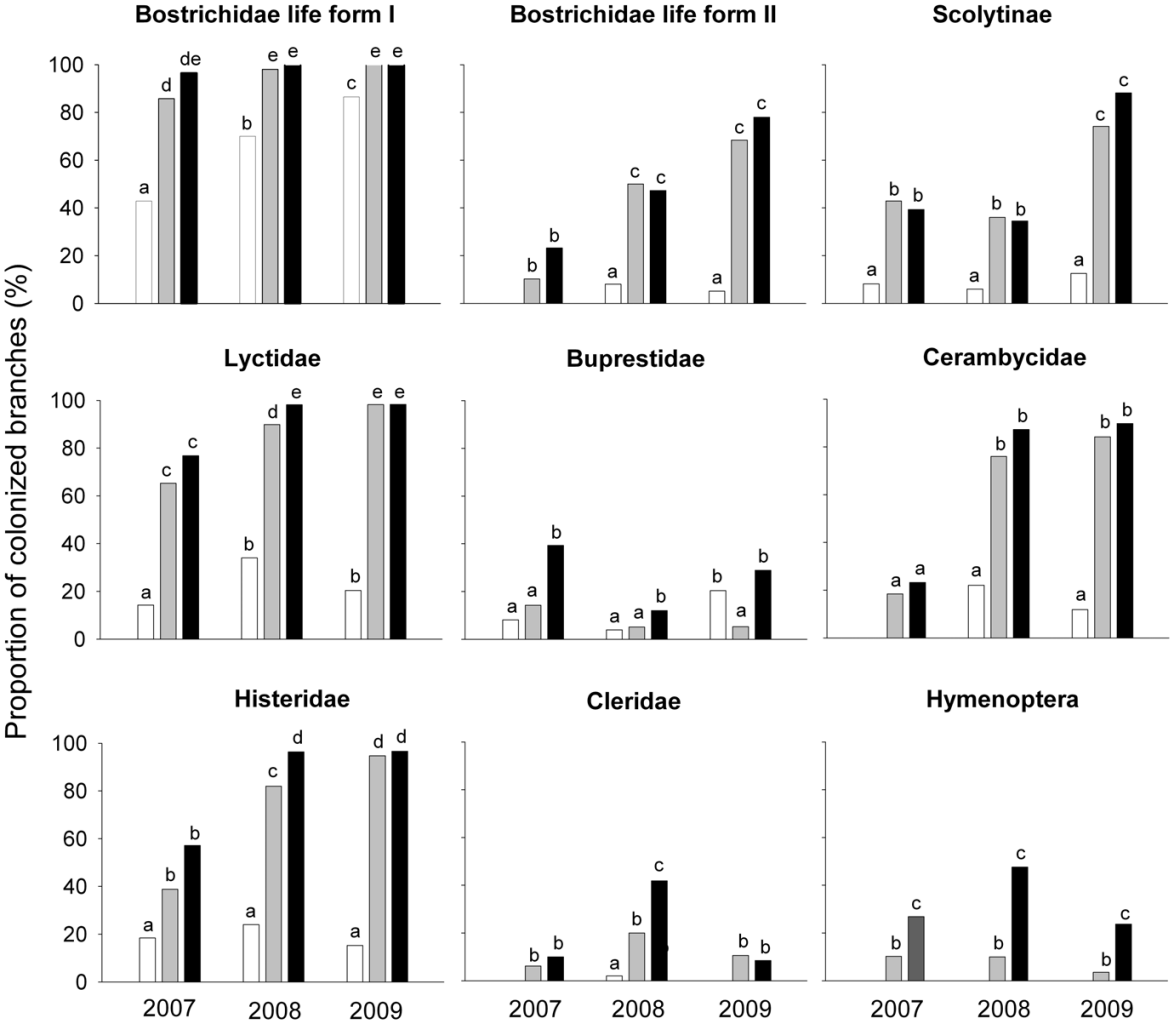
Branch colonization frequency by secondary colonizers. Non-engineered branches are represented by white bars (NE); simulated-engineered branches by gray bars (SE); and *O. albomarginata chamela*- engineered branches by black bars (OE). Values are the percentage of branches colonized. Different letters indicate significant differences (P<0.05) between the frequencies of branch colonization of the treatments.

Data analyses were performed separately by families, with the exception of Bostrichidae, which were analyzed in two species groups because they exhibit two different life forms (I and II; Table 1). The results indicated a highly significant effect of treatment for all families, with the exception of Cleridae; a significant effect of year for all families except for Buprestidae and Hymenoptera; and a significant interaction between treatment and year for Bostrichidae life form II, Lyctidae, Buprestidae and Histeridae (Table 2). All secondary colonizers significantly colonized engineered branches (treatments OE and SE) more frequently than non-engineered branches (NE; Figure 2), with the exception of Buprestidae for which significant differences were found only between *O. albomarginata chamela*-colonized branches (OE) and non-engineered branches (NE) in 2007 and 2008 (Figure 2). The comparison between OE and SE treatments showed variation across years and groups of secondary colonizers (Figure 2), but in general there were no significant effects related to the presence of *O. albomarginata chamela.*

**Table 2:** Effect of habitat engineering on branch colonization frequency by secondary colonizers.

Family	Treatment			Year			Treatment x Year		
	df	F	P	df	F	P	df	F	P
Bostrichidae (Life form I)	2, 307	21.81	<0.0001	2, 307	5.72	0.0036	4, 307	0.47	0.7609
Bostrichidae (Life form II)	2, 259	22.21	<0.0001	2, 259	10.37	<0.0001	4,259	0.1584	<0.0001
Curculionidae (Scolytinae)	2, 307	32.52	<0.0001	2, 307	16.14	<0.0001	4, 307	1.94	0.1040
Lyctidae	2, 307	101.31	<0.0001	2, 307	17.82	<0.0001	4, 307	4.05	0.0032
Buprestidae	2, 307	23.72	<0.0001	2, 307	0.15	0.8619	4, 307	3.30	0.0114
Cerambycidae	2, 259	53.70	<0.0001	2, 259	46.94	<0.0001	4, 259	1.21	0.3078
Histeridae	2, 307	60.73	<0.0001	2, 307	19.55	<0.0001	4, 307	6.48	<0.0001
Cleridae	1, 153	2.73	0.1003	2, 153	9.11	0.0002	2, 153	1.44	0.2408
Hymenoptera	1, 153	16.73	<0.0001	2, 153	1.16	0.3150	2, 153	0.55	0.5762

Data analyses were performed through a generalized linear model with a binomial distribution and a logit link function using a GLIMMIX procedure in SAS.

### Effect of habitat engineering on the abundance of secondary colonizers

**Figure 3:**
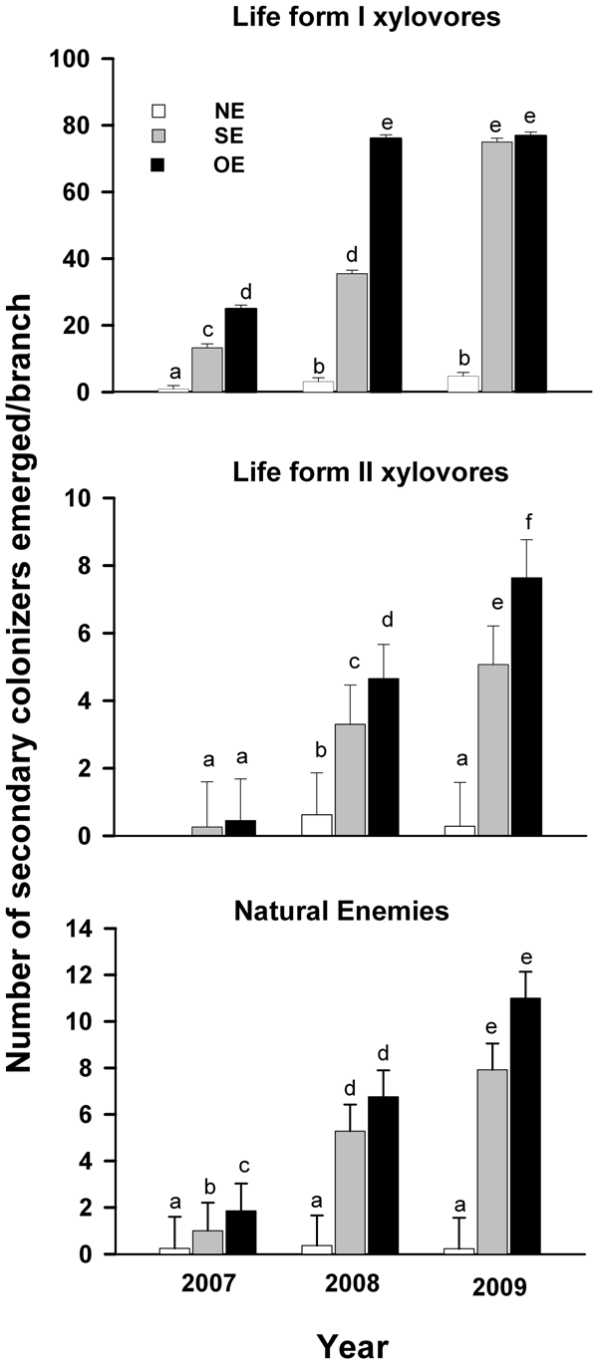
Abundance of secondary colonizers that emerged from *Spondias purpurea* detached branches. Bars indicate LSMeans (±SE) of the number of secondary colonizers that emerged per *S. purpurea* branch in the three studied years. White bars indicate non-engineered branches (NE); gray bars indicate simulated-engineered branches (SE); and black bars indicate *O. albomarginata chamela*-colonized branches (OE). Different letters indicate significant differences between treatments (P<0.05).

There was a highly significant effect of treatment, year and the interaction between treatment and year on the abundance of all groups of secondary colonizers: life form I xylovores (F_2, 319_ = 366.04, P<0.0001; F_2, 319_ = 74.44, P<0.0001; F_4, 475_ = 6.17, P<0.0001); life form II xylovores (F_2, 475_ = 31.34, P<0.0001; F_2, 421_ =  28.54, P<0.0001; F_4, 475_ =  3.21, P = 0.0129); and natural enemies (F_2, 465_ = 127.51, P<0.0001; F_2, 406_ = 30.23, P<0.0001; F_4, 475_ = 5.03, P = 0.0006). The three groups of secondary colonizers showed the following pattern of abundance: OE>SE>NE, where engineered *vs.* non-engineered branches (OE and SE *vs.* NE) showed significant differences for the three groups of secondary colonizers (Figure 3). However, the OE *vs*. SE comparison only showed a significant difference for life form I xylovores in 2007 and 2008, for life form II xylovores in 2008 and 2009, and for natural enemies for 2007 (Figure 3). The abundance of all secondary colonizers in non-engineered branches (NE) was 95% (2007), 93% (2008) and 96% (2009) lower than the abundance of secondary colonizers in *O. albomarginata chamela*-colonized branches (OE) (Figure 3).

### Effect of habitat engineering on the species richness of secondary colonizers

**Figure 4:**
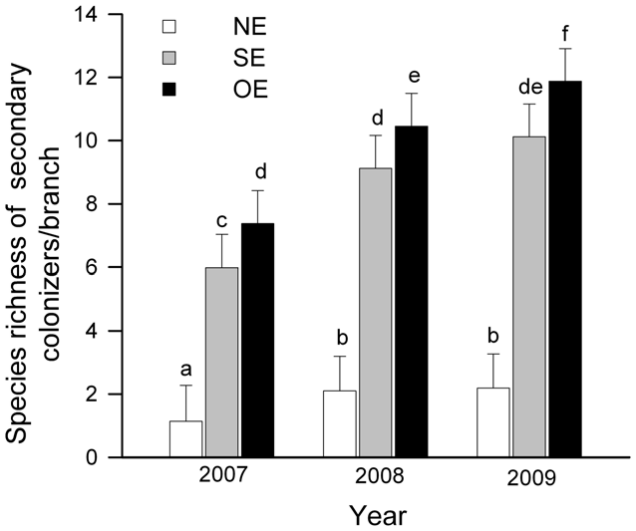
Species richness of secondary colonizers that emerged from *Spondias purpurea* branches. LSMeans (±SE) of the number of species per *S. purpurea* branch in the three studied years; white bars indicate non-engineered branches (NE); gray bars indicate simulated-engineered branches (SE); and black bars indicate *O. albomarginata chamela*-engineered branches (OE). Different letters indicate significant differences between treatments (P<0.05).

**Figure 5:**
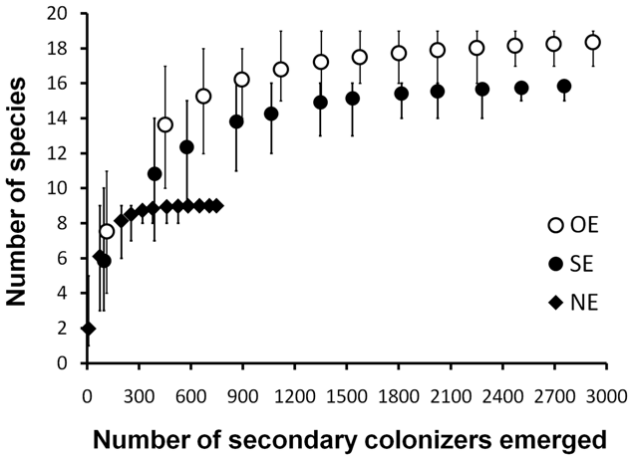
Rarefaction curves plotting the number of species of secondary colonizers *vs.* the number of individuals sampled in detached *Spondias purpurea* branches. NE  =  non-engineered branches; SE =  simulated-engineered branches; and OE =  *O. albomarginata chamela*- engineered branches. Bars represent 95% confidence intervals obtained from 10 000 re-sampling iterations. Bars that overlap the mean for alternate treatments indicate that treatments were not significantly different (P>0.05).

There was a strong effect of treatment (F_2, 478_ = 367.7, P<0.0001), year (F_2, 478_ = 45.77, P<0.0001), habitat engineering (NE *vs.* SE and NE *vs.* OE; Figure 4) and the presence of the ecosystem engineer (OE *vs.* SE; Figure 4) on the species richness of secondary colonizers that emerged from *S. purpurea* branches, with no significant interaction between treatment and year (F_4, 478_ = 0.64, P = 0.6310). The results showed the OE>SE>NE pattern of species richness, consistent across years (Figure 4). NE branches showed 85% (2007), 80% (2008), and 82% (2009) fewer species than OE branches (Figure 4). Rarefaction curves showed that the observed differences in cumulative species richness persisted even when samples were rarefied to similar abundances of individuals (Figure 5).

## Discussion

Several insect herbivores can create new habitats and alter habitat resource availability for other organisms, by modifying the structural and/or nutritional properties of plant tissues [4], [10], [14], [15] **.** *O. albomarginata chamela* actively manipulates its host plant by: (i) girdling and detaching branches, and (ii) gnawing eggs niches and incisions into the bark or stems [27]. These modifications were key factors for the establishment and development of an arthropod community composed by xylovores (Bostrichidae, Scolytinae, Buprestidae, Lyctidae and Cerambycidae) and natural enemies (Histeridae, Cleridae and Hymenoptera).

### Benefits of stem-boring engineering to secondary colonizers

The reported benefits of insect ecosystem engineering to secondary colonizers include: shelter from harsh abiotic factors, avoidance of natural enemies, and modification of resource quality [4], [10], [47]. However, the importance of each benefit differs among insect guilds. For example: leaf rolls and leaf mines are colonized for shelter rather than for the food they contain [11], [15], whereas galls provide shelter, protection from natural enemies and high quality food resources [10].

The main benefits of the stem-boring engineering by *O. albomarginata chamela* to secondary colonizers were related to the creation of a habitat with high availability of oviposition sites, because branches without incisions (non-engineered) were poorly colonized. Moreover, artificially-engineered branches were colonized by a similar arthropod community that colonized branches naturally detached by *O. albomarginata chamela*. This confirms that incisions made by *O. albomarginata chamela* adult females along the detached *S. purpurea* branches are used by other arthropod species as oviposition sites. Availability of oviposition sites offers three benefits to secondary colonizers because they can: (i) save costs of searching for suitable oviposition sites; (ii) diminish the “excavation costs” of the initial stem penetration [10]; and (iii) reduce exophytic predation during the oviposition period (*sensu* [48]).

Additionally, the presence of *O. albomarginata chamela* had a significant impact on the abundance of xylovore species in some years (31–50%), as well as on species richness of the arthropod community in the removed *S. purpurea* branches (15%). The increase of nutrient availability by deposition of faecal pellets is one of the potential benefits of the ecosystem engineer to secondary colonizers [5], [10], [49]. *O. albomarginata chamela* larvae digest cellulose [50], transforming complex structural carbohydrates into simple sugars, which can be eliminated with faecal pellets. Thus, it is possible that this insect supplies partially digested food to secondary colonizers. However, future studies are needed to confirm this hypothesis.

### Effects of stem-boring engineering on arthropod community

Our study demonstrated that ecosystem engineering by *O. albomarginata chamela* had strong positive effects on its associated arthropod community. The abundance and species richness of xylovore insects were higher in engineered branches than in non-engineered branches, possibly due to greater quantity and quality of habitat and food resources provided by engineered branches [51]. This is consistent with previous studies reporting that higher colonization and performance, following the improvement of resource quality, increase the abundance and species richness of insect herbivores [52]–[54]. Another mechanism that promotes high species richness, is the increase of the abundance of rare resources or combinations of resources that are required by specialist species [51], [54], [55]. Five cerambycid species were restricted to engineered branches in the three studied years (Table 1). These species were among the larger xylovore colonizers (Life form II; Table 1). Larger insect species produce larger eggs [44], indicating that they require larger egg-niches. Species of the genus *Oncideres* gnaw large egg niches (4–5 mm in width) in which oviposit [17], whereas cerambycid species that do not have the ability to gnaw egg niches, wander over the hosts probing the bark with the ovipositor for cracks and crevices in which they oviposit [30]. Therefore, it is possible that the size of egg niches gnawed by *O. albomarginata chamela* females allow these species to oviposit in them. These findings suggest that the increased species richness in engineered branches can be a consequence of the greater abundance of specific egg niches required by specialist species.

The increase in abundance and number of secondary xylovores, which represent potential prey and hosts for natural enemies, in turn may influence the abundance and species richness of natural enemies and result in bottom-up effects [3], [4]. In this study, the overall abundance and species richness of natural enemies was higher in engineered branches than in non-engineered branches. Specifically, only one (Histeridae: *Teretriosoma nigrescens*) of the three natural enemies was consistently recorded in non-engineered branches (Figure 2). *T. nigrescens* preys upon some bostrichid beetles [56], which were the main species that colonized non-engineered branches. However, there were five cerambycid species that did not colonize non-engineered branches. Parasitic wasps are reported as one of the main natural enemies of cerambycid beetles [57]. Thus, the absence of colonization of parasitic wasps in non-engineered branches could be related to the reduced colonization by cerambycid beetles.

Our results confirm the notion that changes in the composition of the xylovore community cascade upward to higher trophic levels through bottom-up effects.

### Implications of stem-boring ecosystem engineering for biodiversity

On average, ecosystem engineering by *O. albomarginata chamela* was responsible for nearly 95% of the abundance of secondary colonizers and 82% of the species richness. These results are consistent with the positive effects on arthropod diversity reported for other insect ecosystem engineers [11]–[13], [15]. However, ecosystem engineering by *O. albomarginata chamela* had greater effects on species richness than leaf-roller caterpillars (14–84%) [11], [12], gall-makers (32%) [13], and leaf-miners [15], possibly because ecosystem engineering by this species allowed the establishment of an entire arthropod community, and regulated the structure of this community. Therefore, based on Painés “keystone” concept [58], ecosystem engineering by *O. albomarginata chamela* can be considered a keystone process (*sensu* [7]).

There are two explanations for this keystone process: the existence of a highly structured community, and the degree of specialization (i.e. interaction strength) between the secondary colonizers and the engineered habitat [58]. The arthropod community associated with branches engineered by *O. albomarginata chamela* is a highly structured community, because it consists of organisms with different life history traits and trophic positions. In addition, our study suggests that the xylovore community associated with *S. purpurea* branches might be specialists in branches girdled and detached by *O. albomarginata chamela*. Furthermore, the known host plants for the Cerambycidae and Scolytinae species emerging from *S. purpurea* branches completely correspond to the alternate host plants of *O. albomarginata chamela,* and to the host plants of other girdling-beetles in the study site, such as *Oncideres rubra* and *Taricanus zaragozai* [32], [59]. Some of these cerambycid species, as well as most species of Bostrichidae and Buprestidae in the *S. purpurea* branches, also use branches girdled by other beetle species in different tropical and subtropical regions [17], [18], [20], [60].

### Conclusions

The importance of interactions mediated by insects in shaping herbivore communities is becoming widely recognized [4]. However most studies have focused on herbivore-induced changes to plant chemical composition (reviewed in [4]) and only few to plant-structural modifications made by insect engineers (reviewed in [10]). This study provides evidence that interactions mediated by ecosystem engineering may be a common factor enhancing species richness and structuring communities of borer insects. Therefore, our findings have important implications for conservation, because through the understanding of the mechanisms underlying ecosystem engineering it is possible to develop effective strategies of ecosystem management [61].
